# Risk factors of postoperative bone cement leakage on osteoporotic vertebral compression fracture: a retrospective study

**DOI:** 10.1186/s13018-021-02337-1

**Published:** 2021-03-10

**Authors:** Kui Zhang, Jiang She, Yandong Zhu, Wenji Wang, Erliang Li, Ding Ma

**Affiliations:** 1 Department of Orthopedics, Ninth Hospital of Xi’An, Xi’An, 710000 ShaanXi China; 2 Department of Orthopedics, The First Clinical Medical College of Lanzhou University, Lanzhou, 730000 China; 3 Department of Orthopedics, Tangdu Hospital, Airforce Medical University, Xi’An, 710000 ShaanXi China

**Keywords:** Osteoporotic vertebral compression fracture, Cement leakage, Percutaneous vertebroplasty, Risk factors

## Abstract

**Purpose:**

To investigate risk factors of bone cement leakage in percutaneous vertebroplasty(PVP)for osteoporotic vertebral compression fracture (OVCF).

**Methods:**

A total of 236 patients (344 vertebrae) who underwent PVP between November 2016 and June 2020 were enrolled in the study. Clinical and radiological characteristics, including age, gender, course of disease, trauma, type of vertebral fracture, cortical continuity of vertebral body, intervertebral vacuum cleft (IVC), fracture severity, fracture level, basivertebral foramen, bone cement dispersion types, the cement injection volume, the type of cement leakage, puncture approach, and intrusion of the posterior wall, were considered as potential risk factors. Three types of leakage (type-B, type-C, and type-S) were defined and risk factors for each type were analyzed. Logistic analysis was used to study the relationship between each factor and the type of cement leakage.

**Results:**

The incidences of the three types of leakage were 28.5%, 24.4%, and 34.3%. The multinomial logistic analysis revealed that the factors of type-B leakage were the shape of cement and basivertebral foramen. One significant factor related to type-C leakage was cortical disruption, and the factors of type-S leakage were bone cement dispersion types, basivertebral foramen, cleft, fracture severity, an intrusion of the posterior wall, and gender.

**Conclusion:**

Different types of cement leakage have their own risk factors, and the analysis of risk factors of these might be helpful in reducing the rate of cement leakage.

**Supplementary Information:**

The online version contains supplementary material available at 10.1186/s13018-021-02337-1.

## Introduction

With the aging population growing, the incidence of osteoporotic vertebral compression fracture has risen in the world’s common diseases [1, 2]. A report reveals about the incidence of vertebral fracture of the elderly population was 8.7% for men in Japan in 2020 [1]. In Thailand, one study in postmenopausal Thai women showed that the prevalence rate of vertebral fracture was 29% in 2020 [2]. The clinical manifestations of OVCF are mainly the mobility decreases, reduced pulmonary function, higher risk of bed-related complications, and higher mortality rate [3]. PVP is a safe and effective treatment for OVCF. Klazen et al. [3] reported that the PVP group had fewer serious complications or adverse and significant improvement in the quality of life. The most frequent complication of PVP is bone cement leakage [4]. For patients with heart disease, liver and kidney dysfunction, and poor tolerance, the risk of major complications related to cement leakage (pulmonary embolism, etc.) may be larger. When cement leaks into the spinal canal or nerve root canal, it could lead to neurological complications such as paraplegia and nerve root compression, although most of these are asymptomatic. The cement can also leak into the blood vessel, causing pulmonary embolism that is lethal [5]. Early recognition of those risk factors and prompt treatment is essential to prevent devastating sequelae and make a balanced treatment decision.

In view of this, we conducted this retrospective study of 236 patients (344 vertebrae) OVCF who underwent PVP to explore possible risk factors for bone cement leakage and provide more sufficient theoretical basis support to reduce bone cement leakage risk in PVP.

## Data and methods

### General information

Between November 2016 and June 2020, we performed PVP on a total of 236 patients with OVCF. A total of 344 vertebrae were treated.

Patients were required to meet the following inclusion criteria: (1) patients with OVCF, (2) back pain that related by radio-logical examination lasting at least 2 months with no relief of symptoms with conservative treatment, (3)complete clinical data, and (4) PVP performed for the first time. Patients were required to meet the following exclusion criteria: (1) pathological vertebral compression fracture caused by multiple myeloma, spinal metastases, etc; (2) secondary osteoporosis; (3) abnormal blood coagulation and cardiopulmonary function cannot tolerate surgery; and (4) vertebral compression bone is traumatic.

All operations were expected to be completed by Dr. Ma, an experienced spine surgeon. Patients were invited to in the prone position for the procedure. The needle that was removed from the trochar, a proper amount of cement, was injected into the vertebral body with the guidance of a C-arm X-ray machine (SIEMENS, Germany). After the administration of local anesthesia with 1% lidocaine, 10- or 11-gauge needles (KINETIC, China) were unilaterally or bilaterally inserted into the affected vertebral body transpedicularly. A unilateral approach was preferable if only one side of the neurologic element was compressed by the fractured vertebral body; a bilateral approach was frequently used when the distribution of cement was unsatisfactory or asymmetrical. After the placement of needles was hammered into the anterior third of the vertebral body, with X-ray guidance, bone cement (polymenthylmethacrylate, StrykexP, USA) was gently rapidly injected into the fractured vertebral body. Cement was admitted between 4 and 8 minutes until it arrives “tooth-paste-like” phase, to reduce the risk of extravasation. The amount of bone cement injected was 1.5~9.0 ml.

### Observation indicators

To determine the risk factors for bone cement leakage after PVP, clinical information and characteristics were collected, including age, gender, course of disease, trauma, type of vertebral fracture, cortical continuity of vertebral body, intervertebral vacuum cleft, fracture severity, fracture level, basivertebral foramen, bone cement dispersion types, the cement injection volume, the type of cement leakage, puncture approach, and intrusion of the posterior wall, and were considered as potential risk factors.

According to the duration of the disease, we divided the disease into three categories: acute (<2 weeks), subacute (2 weeks ~ 2 months), and chronic (>2 months). The discontinuous cortical bone is defined that local discontinuity that occurred in the low-signal bone cortex on MR I[6] or interruption of local continuity of the bone cortex in CT. The location of the interruption of cortical continuity can be found in front, both sides, upper and lower endplates, and rear of the vertebral body. IVC criteria were as follows: the permeable fissures on X-ray or CT are located in the center of or near the upper and lower endplates of the vertebral body; the fissures on MRI are usually abnormal, well-defined, linear or cystic low-signal intensity in the vertebral body, similar to air, and T2 images show high- or low-signal intensity [7]. The types of vertebral fracture include wedge-shaped fracture, biconcave-shaped fracture, and a fracture with compression [8]. The intrusion of the posterior wall refers to the part of the posterior wall of the vertebral body that protrudes into the spinal canal on the axial CT image [9].

Basing on the semi-quantitative method of Genant using lateral X-ray, fracture severity was classified as mild, moderate, and severe. It defined as reductions in the anterior, middle, or posterior height of 20–25%, 26–40%, and >40%, respectively [8].

Vertebral fractures were marked with spine X-rays according to the semi-quantitative method. Vertebrae were graded as normal, or with mild, moderate or severe deformities, defined as reductions in the anterior, middle, or posterior height of 20–25%, 25–40%, and >40%, respectively. The vertebrobasilar venous foramen refers that when the vertebrobasilar vein passes the center of the vertebral body posterior wall, the bone defect which is shown as triangular or irregular quadrilateral on CT sagittal position, or the porous defect of the bone is shown on CT axial position [10]. According to the bone cement dispersion types, patients were subdivided into the mass type and diffusion type [11]. Tsai et al. [12] have classified the leaks of the cement into three types: those via the basivertebral vein (type-B), via the segmental vein (type-S), and through a cortical defect (type-C, a special type). Precise quantitative assignments and results of the above clinical-related factors are shown in Tables 1 and 2.

**Table 1:** Factors related to cement leakage and their assignment

	Assignment
Age	≤85 years (1); >85 years (2)
Gender	Male (1); female (2)
Course of disease	Acute (1); subacute (2); chronic (3)
Trauma	No (1); yes (2)
Fracture level	Mid-thoracic (1); Low thoracic (2); Lumbar (3)
Cortical continuity of vertebral body	No (1); yes (2)
Intervertebral vacuum cleft	No (1); yes (2)
Type of vertebral fracture	Wedge (1); biconcave (2); crush (3)
Fracture severity	Mild (1); moderate (2); severe (3)
Intrusion of posterior wall	No (1); yes (2)
Basivertebral foramen	No (1); yes (2)
Puncture approach	Unilateral (1); bilateral (2)
Bone cement dispersion types	Cleft (1); trabecular (2)
Cement volume	<6.0 ml (1); ≥6.0 ml (2)

**Table 2:** Results of assignment on each group

	Type-B leakage	Others	Type-C leakage	Others	Type-S leakage	Others
Age	1 (66); 2 (32)	1 (161); 2 (85)	1 (60); 2 (24)	1 (195); 2 (65)	1 (88); 2 (30)	1 (167); 2 (59)
Gender	1 (20); 2 (78)	1 (77) 2; (169)	1 (26); 2 (58)	1 (71); 2 (189)	1 (42); 2 (76)	1 (55); 2 (171)
Course of disease	1 (76); 2 (15); 3 (7)	1 (195); 2 (41); 3 (10)	1 (65); 2 (15); 3 (4)	1 (206); 2 (41); 3 (13)	1 (98); 2 (17); 3 (3)	1 (173); 2 (39); 3 (14)
Trauma	1 (60); 2 (38)	1 (122); 2 (124)	1 (42); 2 (42)	1 (140); 2 (120)	1 (64); 2 (54)	1 (118); 2 (108)
Fracture level	1 (9); 2 (37); 3 (52)	1 (26); 2 (87); 3 (133)	1 (7); 2 (37); 3 (40)	1 (28); 2 (87); 3 (145)	1 (10); 2 (38); 3 (70)	1 (25); 2 (86); 3 (115)
Cortical continuity of vertebral body	1 (51); 2 (47)	1 (112); 2 (134)	1 (3); 2 (81)	1 (160); 2 (100)	1 (67); 2 (51)	1 (96); 2 (130)
Intervertebral vacuum cleft	1 (79); 2 (19)	1 (160); 2 (86)	1 (39); 2 (45)	1 (200); 2 (60)	1 (104); 2 (14)	1 (135); 2 (91)
Type of vertebral fracture	1 (55); 2 (15); 3 (28)	1 (134); 2 (27); 3 (85)	1 (35); 2 (12); 3 (37)	1 (154); 2 (30); 3 (76)	1 (79); 2 (13); 3 (26)	1 (110); 2 (29); 3 (87)
Fracture severity	1 (72); 2 (19); 3 (7)	1 (164); 2 (53); 3 (29)	1 (50); 2 (19); 3 (15)	1 (186); 2 (53); 3 (21)	1 (98); 2 (18); 3 (2)	1 (138); 2 (54); 3 (34)
Intrusion of posterior wall	1 (68); 2 (30)	1 (159); 2 (87)	1 (40); 2 (44)	1 (187); 2 (73)	1 (96); 2 (22)	1 (131); 2 (95)
Basivertebral foramen	1 (58); 2 (40)	1 (191); 2 (55)	1 (61); 2 (23)	1 (188); 2 (72)	1 (73); 2 (45)	1 (176); 2 (50)
Puncture approach	1 (5); 2 (93)	1 (12); 2 (234)	1 (2); 2 (82)	1 (15); 2 (245)	1 (5); 2 (113)	1 (12); 2 (214)
Bone cement dispersion types	1 (13); 2 (85)	1 (92); 2 (154)	1 (40); 2 (44)	1 (65); 2 (195)	1 (15); 2 (103)	1 (90); 2 (136)
Cement volume	1 (62); 2 (36)	1 (169); 2 (77)	1 (53); 2 (31)	1 (178); 2 (82)	1 (74); 2 (44)	1 (157); 2 (69)

### Statistical methods

Statistical Packages for Social Sciences V20.0 (SPSS, V20.0; IBM Corporation, NY, USA) was used to analyze the data, and univariate analysis using chi-squared test or the nonparametric Wilcoxon test for categorical and continuous variables was used to identify risk factors. We used multivariate logistic analysis to provide a prediction model to predict bone cement leakage. Multinomial logistic analysis was performed using a stepwise approach to determine independent predictors of the occurrence of each type of cement leakage. *P* values <0.05 were regarded as significant.

## Results

A total of 236 patients (75 males and 161 females) with 344 treated vertebrae were contained in our study. The mean patient age was (83.9±3.2) years (range, 79~95 years). The average duration of disease was (12.8±17.7) days (range, 2~119 days). Among the 344 vertebrae studied, 159 (46.2%) were thoracic, and 185 (53. 78%) were lumbar. The fracture caused by no definite trauma was 182 vertebrae, and the fracture caused by definite trauma was 162 vertebrae. Seventeen vertebrae underwent unilateral pedicle puncture and 327 vertebrae underwent bilateral puncture way. The most common type of leakage was type-S, with 118 cases (34.3% of all cases). The other two types were type-B (98 cases, 28.5%), type-C (84 cases, 24.4%).

Univariate analysis showed that gender, IVC, and the bone cement dispersion types had a significance with type-B cement leakage after PVP (*P* < 0.05), that the discontinuous cortical bone, IVC, type of vertebral fracture, fracture severity, intrusion of the posterior wall, and the bone cement dispersion types were had a significant with type-C cement leakage (*P* < 0.05), that gender, the discontinuous cortical bone, IVC, type of vertebral fracture, fracture severity, intrusion of posterior wall, the bone cement dispersion types, and the vertebrobasilar venous foramen were had a significant type-S with cement leakage (*P* < 0.05) (Table 3).

**Table 3:** Univariate analysis of potential risk factors for occurrence of each type cement leakage

	Type-B leakage patients vs. others		Type-C leakage patients vs. others		Type-S leakage patients vs. others	
	*X*^*2*^	*P*	*X*^*2*^	*P*	*X*^*2*^	*P*
Age	3.286	0.070	0.422	0.516	0.019	0.891
Gender	4.107	0.043	0.417	0.519	4.852	0.028
Course of disease	1.449	0.484	0.205	0.903	2.895	0.235
Trauma	3.805	0.051	0.377	0.539	0.128	0.721
Fracture level	0.256	0.880	3.128	0.209	2.272	0.321
Cortical continuity of vertebral body	1.192	0.275	85.567	0.000	6.360	0.012
Intervertebral vacuum cleft	8.013	0.005	27.840	0.000	29.487	0.000
Type of vertebral fracture	1.847	0.392	8.200	0.017	11.318	0.003
Fracture severity	2.074	0.355	7.290	0.026	21.429	0.00
Intrusion of posterior wall	0.706	0.401	16.709	0.000	18.899	0.000
Basivertebral foramen	11.945	0.001	0.003	0.956	9.943	0.002
Puncture approach	0.007	0.931	1.552	0.213	0.190	0.190
Bone cement dispersion types	19.247	0.00	15.317	0.000	26.869	0.000
Cement volume	0.938	0.333	0.829	0.363	1.605	0.205

A multinomial logistic regression analysis was conducted to identify the risk factors for each of the three types of cement leakage (Table 4). The vertebrobasilar venous foramen and the bone cement dispersion types (diffusion type) (*P* < 0.05) were found to be significant and strong predictors of type-B cement leakage. The discontinuous cortical bone was a strong predictor of type-C bone cement leakage. (surgical approach) (*P* < 0.05). Bone cement dispersion types (diffusion type) and the vertebrobasilar venous foramen were found to be strong and significant predictors of type-S cement leakage. However, IVC (OR=0.335), fracture severity, intrusion of the posterior wall (OR=0.487), and gender (female) (OR=0.425) were the protective factors of type-S bone cement leakage (Table 4).

**Table 4:** Multivariate analysis of risk factors for occurrence of each type cement leakage

	OR	*P*	95%CI
Type-B leakage			
Gender	1.589	0.121	0.885–2.854
Intervertebral vacuum cleft	0.682	0.218	0.371–1.254
Basivertebral foramen	2.364	0.001	1.398–3.996
Bone cement dispersion types	3.443	0.000	1.759–6.740
Type-C leakage			
Cortical continuity of vertebral body	35.183	0.000	10.554–117.288
Intervertebral vacuum cleft	1.352	0.369	0.700–2.611
Type of vertebral fracture	1.023	0.909	0.697–1.500
Fracture severity	0.874	0.584	0.540–1.416
Intrusion of posterior wall	1.258	0.491	0.655–2.419
Bone cement dispersion types	0.562	0.078	0.296–1.066
Type-S leakage			
Gender	0.425	0.004	0.239–0.756
Cortical continuity of vertebral body	1.004	0.990	0.578–1.743
Intervertebral vacuum cleft	0.335	0.003	0.164–0.682
Type of vertebral fracture	1.006	0.971	0.715–1.417
Fracture severity	0.514	0.015	0.300–0.880
Intrusion of posterior wall	0.487	0.025	0.260–0.916
Basivertebral foramen	2.272	0.003	1.313–3.931
Bone cement dispersion types	3.548	0.000	1.822–6.909

## Discuss

### Characteristics of elderly patients

The elderly patients exhibit several unique disease characteristics. The main results are the following: (1) recent evidence highlights patients with cardiopulmonary disease as the elderly patient group. Clarencon et al. [13] study shows that about 86.5% of elderly patients had cardiovascular risk factors (hypertension, hypercholesterolemia, venous insufficiency, arrhythmia, diabetes); about 10.0% of the patients were complicated with lung diseases (chronic bronchopneumonia, asthma, etc.). (2) There was no obvious history of traumatic fracture for elderly patients. In our study, among the OVCF patients treated in our hospital. The proportion of patients with no history of trauma was 47.1% (162/344). (3) There is a higher proportion of IVC. Nieuwenhuijse et al. [14] and Ding et al. [15] found in a group of cohorts with an average age of 73.2 and 69.4 years, respectively, that the IVC accounted for 18.1% (32/177) and 18.2% (53/292). In our study, the average age was 83.9±3.2 years old, and the vertebral body with IVC accounted for 30.5% (105/344). (4) Fracture severity does not necessarily reflect more severe. In people over 80 years old, regardless of the fact that the BMD is lower, and the incidence of trauma is relatively lower. Young et al. [16] reported that patients over 80 years old were significantly more than those under 80 years old in severe vertebral compression (over 2/3 height of vertebra), but Liang et al. [17] reported that patients very elderly patients (≥80 years old) were more likely to undergo a repeat vertebral augmentation (15.2%) than were those patients in other age groups (8.0–10.9%).

### Effect of vertebroplasty in elderly patients

There have been many studies on the safety and efficacy of vertebroplasty in elderly patients with OVCFs [13, 18, 19]. They all concluded that vertebroplasty is a safe and effective surgical procedure for the treatment of OVCFs. However, no major complications such as pulmonary embolism, spinal cord injury, and nerve root injury were noted in these studies. Uemura et al. [18] and Kaufmann et al. [19] reported that the changes in blood pressure, heart rate, and blood oxygen saturation were observed, and it was found that there was a significant correlation between bone cement and cardiopulmonary dysfunction.

Some cohort studies and systematic review studies show that the leakage rates of type-B, type-C, and type-S are 21.3%, 50.9%, and 11.0%, respectively [4, 14, 15]. In our research which was similar to the above study, rates were 28.5%, 24.4%, and 34.3%, respectively. Some researchers have analyzed the risk factors of leakage or a certain type of leakage. The data in these studies are quite heterogeneous [4, 14, 20]. In our study, the risk factors of type-B, type-C, and type-S leakage were reported, respectively.

1. Bone cement dispersion-type is diffuse, which is a risk factor for types B and S. Most scholars focus on the relationship between bone cement morphology and recurrent fracture after the operation of injured vertebrae [21]. However, there are a lack of reports on the relationship between bone cement morphology and bone cement leakage [22]. There are venous channels between the trabeculae, which crisscross and converge to form the vertebrobasilar venous system, and the dispersed bone cement is easy to enter into the channel to form type-B and type-S leakage. The shape of bone cement in the vertebral body is related to numerous factors, such as trabecular density, bone cement pressure, bone cement viscosity, and cement content [23].
2. Vertebrobasilar venous foramen which was shown on CT is the risk factor for type B and S leakage. It is linked to many venous channels in the vertebral body, so the blood can flow in both directions, drained backward to the intraspinal venous plexus (the anatomical basis of type-B leakage) and forward to the extravertebral venous plexus (the anatomical basis of type S leakage) [12]. The vertebrobasilar venous foramen was not displayed on CT. The author estimated that there may be the following reasons: ① the posterior wall of the vertebral body fracture destroyed the anatomical structure. One study showed that in sagittal burst vertebral body fractures, the probability of fracture location involving this area is about 50.0%; the probability of posterior wall fractures involving this area is about 90% [24]. ② Ischemic osteonecrosis occurred in the fractured vertebral body. When the vertebral body fissures or collapses, the veins in the vertebral body can be secondary damaged, closed, or disappeared. At present, there is not any report on the correlation between type-S cement leakage and vertebrobasilar venous foramen. The author speculates that the display of the vertebrobasilar venous foramen on CT may indicate that the vertebrobasilar vein is intact. It is best for the vertebral body along the intervertebral vein, that is, the anatomical basis of the leakage of bone cement through the anterior segmental vein of the vertebral body is good, so the risk of type-S leakage is increased.
3. Cortical disruption of the vertebral body is a risk factor for type-C leakage (discontinuity of the bone cortex of the vertebral body), which has been recognized by most scholars [4, 6, 14]. Tomé-Bermejo et al. [4] considered that the independent risk factors for this type of leakage were fracture severity and fracture type. Ding et al. [15] concluded that intrusion of posterior wall, fracture severity, IVC, and the bone cement dispersion types. According to the subgroup data of our study (intervertebral disc leakage rate 12.5%, 43/344), multivariate regression analysis showed that there was just one independent risk factor for intervertebral disc leakage, that is, interruption of vertebral cortical continuity. This conclusion is theoretically consistent with clinical practice, that is, in addition to iatrogenic injuries (such as puncture needle piercing the upper or lower endplate cortex) [20]. In our study, the leakage of 3 vertebrae may be due to iatrogenic injury during operation.
4. The intrusion of the posterior wall, fracture severity, IVC, and gender (woman) is the protective factors of type-S leakage. Scholars have not reached a consistent conclusion on the correlation between IVC and type-S leakage [4, 14, 15]. Ding et al. [15] and Nieuwenhuijse et al. [14], in a cohort study with an average age of 69.4 years and 73.2 years, respectively, showed that IVC was not a protective factor for type-S bone cement leakage by multivariate regression analysis. Li et al. [25] discovered that the older the age and the lower the bone mineral density, the higher the probability of fissure sign. The average age of the patients in our study was 83.9±3.2 years old, and the bone mineral density may be low and the proportion of fissure signs higher. Therefore, the author speculates that one of the reasons why the above two scholars’ conclusions are different from ours. Tome-Bermejo et al. [4] believe that IVC increases the uniformity and controllability of the cement filling process due to the dead space in the vertebral body, which is similar to the principle of percutaneous kyphoplasty, thus reducing the risk of type-S leakage (segmental venous leakage). The authors believe that the presence of IVC during the filling of bone cement reduces the pressure in the vertebral body, thus reducing the risks of leakage of bone cement into the segmental vein. The cone containing IVC reduces the pressure in the cone during cement filling, thus reducing the risk of cement leakage to segmental veins.

With regard to the study on the correlation between the degree of fracture and type-S leakage, our study is basically consistent with other scholars [4, 14, 15], that is, the more severe the fracture, the more serious the vascular damage of the vertebral venous system. The lower the risk of leakage of bone cement through the vertebral basal vein and forward to the extra-vertebral vein.

There is as yet a lack of literature data on the correlation between intrusion of the posterior wall, gender, and type-S leakage. Our study shows that there is a significant correlation between the two groups, but the cause and mechanism are not clear.

## Conclusion

The vertebrobasilar venous foramen and the bone cement dispersion types (diffusion type) were found to be significant and strong predictors of type-B cement leakage. The discontinuous cortical bone was a strong predictor of type-C bone cement leakage. Surgical approach, bone cement dispersion types (diffusion type), and the vertebrobasilar venous foramen were found to be strong and significant predictors of type-S cement leakage. However, IVC, fracture severity, intrusion of the posterior wall, and gender (female) were the protective factors of type-S bone cement leakage.

In conclusion, clinicians should pay full attention to the OVCF patients whose age is ≥80 years old and who have various cardiopulmonary diseases. The analysis of the risk factors of bone cement leakage is helpful to take targeted measures to decrease the incidence of leakage and its related complications. Our study is based on the conclusion of people over 80 years old. For patients<80 years old, whether the rate of bone cement leakage and the independent risk factors of leakage is similar to our study, it needs further clinical comparative research. There are some additional shortcomings in our study: retrospective study, information bias, single-center study, and small sample size.

## Supplementary Information

**Additional file 1.**

## Data Availability

The data used and/or analyzed during the current study are available from the corresponding author on reasonable request.

## Abbreviations

PVP: Percutaneous vertebroplasty
IVC: Intervertebral vacuum cleft
OVCF: Osteoporotic vertebral compression fracture
CT: Computed tomography
SPSS: Statistical Packages for Social Sciences
